# Mapping the diversity of land uses following deforestation across Africa

**DOI:** 10.1038/s41598-024-52138-9

**Published:** 2024-01-19

**Authors:** Robert N. Masolele, Diego Marcos, Veronique De Sy, Itohan-Osa Abu, Jan Verbesselt, Johannes Reiche, Martin Herold

**Affiliations:** 1 Laboratory of Geo-Information Science and Remote Sensing, Wageningen University and Research, Droevendaalsesteeg 3, 6708 Wageningen, PB The Netherlands; 2 Inria, University of Montpellier, Montpellier, France; 3 Department of Remote Sensing, Julius-Maximilians-University, Oswald-külpe-Weg, 97074 Würzburg, Bayern Germany; 4 GFZ, German GeoResearch Center, Potsdam, Germany

**Keywords:** Climate change, Computational science, Scientific data, Forestry, Environmental impact

## Abstract

African forest are increasingly in decline as a result of land-use conversion due to human activities. However, a consistent and detailed characterization and mapping of land-use change that results in forest loss is not available at the spatial-temporal resolution and thematic levels suitable for decision-making at the local and regional scales; so far they have only been provided on coarser scales and restricted to humid forests. Here we present the first high-resolution (5 m) and continental-scale mapping of land use following deforestation in Africa, which covers an estimated 13.85% of the global forest area, including humid and dry forests. We use reference data for 15 different land-use types from 30 countries and implement an active learning framework to train a deep learning model for predicting land-use following deforestation with an F1-score of $84\pm 0.7$ for the whole of Africa. Our results show that the causes of forest loss vary by region. In general, small-scale cropland is the dominant driver of forest loss in Africa, with hotspots in Madagascar and DRC. In addition, commodity crops such as cacao, oil palm, and rubber are the dominant drivers of forest loss in the humid forests of western and central Africa, forming an *“arc of commodity crops”* in that region. At the same time, the hotspots for cashew are found to increasingly dominate in the dry forests of both western and south-eastern Africa, while larger hotspots for large-scale croplands were found in Nigeria and Zambia. The increased expansion of cacao, cashew, oil palm, rubber, and large-scale croplands observed in humid and dry forests of western and south-eastern Africa suggests they are vulnerable to future land-use changes by commodity crops, thus creating challenges for achieving the zero deforestation supply chains, support REDD+ initiatives, and towards sustainable development goals.

## Introduction

Understanding the dynamics of land-use following deforestation is an important step in climate change mitigation, having a significant effect on forest biomass, biodiversity, and the water cycle^1^. Over the past two decades, Africa has been experiencing a rapid decline in its forest cover or tree cover^2^. Here we define forest cover or tree cover as adopted from ^2^ as all vegetation taller than 5 meters in height.The implications of which is the decline in species richness, changes in the water cycle, and loss of forest carbon stock^3^. The complexion of these changes can vary depending on the location, intensity, and spatial extent of forest loss. Thus understanding the spatial-temporal extent and patterns of the drivers of forest loss in Africa is crucial to comprehending its negative impacts on the forest ecosystem and its contribution to greenhouse gas emissions.

Previous studies suggest agriculture-related land-use change as Africa’s main cause of deforestation^4^. However, such information is derived from courser thematic maps or sample-based land-use statistics^5^. There is a lack of thematically detailed data both at small and large-scale to link deforestation to its respective drivers^6^. Specifically, the much-needed and highly detailed thematic information on the types of direct drivers causing deforestation in Africa is rarely observed in the literature, except for a few countries (mainly in the tropical humid forests)^5,7^. The availability of large-scale, thematic detailed, and high-resolution maps of land use following deforestation in Africa is essential for strategic planning and implementation of deforestation mitigation actions by governments and forest protection agencies^8^.

In addition, most studies focus their driver assessment on the knowledge of localizing specific drivers in specific regions or countries. For example, the latest studies projected an expansion of oil palm, cacao farms in west Africa^9,10^, and small-scale agriculture in the Democratic Republic of Congo^7^. The generally accepted view of forest conversion in Africa aligns with the historical expectation of persistence of various states of subsistence agriculture activities, but little conversion to newly introduced or other types of land uses not previously found in the current location^11^. Aligning with the general view that certain land uses are restricted in certain geographical locations, thus overlooking the diverse actual causes of forest loss critical for deforestation monitoring and mitigation efforts at both local and regional scales.

Despite the availability of national or regional statistics to document the trends of forest loss, consistent, detailed, spatially explicit estimates of the continent’s drivers of forest loss area are lacking^2,12–14^. Many efforts to map drivers of forest loss rely on expert-based visual interpretation from samples of high-resolution satellite images^7,15^. However, the limitation of expert/sample-based visual interpretation is that it lacks detail and consistency in identifying and mapping the drivers over large regions and across time. This, in turn, causes a broad generalization of the drivers of forest loss and, thus, misses most areas and limits the level of detail in pinpointing the exact causes of forest loss useful for government agencies (local, national) responsible for forest monitoring^4^. For example, although the latest study of forest change in tropical forests purportedly assessed agriculture expansion over space and time, its delineation of land uses largely relied on sample-based single-date expert interpretation, and the wall-to-wall-map was not reported^5^.

The absence of detailed systematic monitoring of forest loss drivers complicates assessments of REDD+ efforts and net zero commitments on reducing the impacts of land-use change on forest ecosystems^4,8,13,15^. Generalization and confusion between land-use and land-cover change maps produced at a global scale lead to ‘cryptic forest loss’ and/or overestimates certain land-use and land-cover^4^. This is especially true in Africa, with its rapid deforestation rates and diverse land uses^2,16^. National reporting indicates a substantial net increase in forest loss^16^, but keeping track of net changes in the area of land-cover types that undergo frequent disturbances, like forest regrowth, can lead to the underestimation of the occurrence of tree crop expansion as, with time, they become confused or resemble forest cover^16,17^. This is especially true in dry forests where tree crops (i.e., cashew) resemble natural forest trees (i.e., Miombo)^2^. Thus, it remains unclear whether natural forest recovery or tree crops are driving expansions in forest cover in Africa. Specifically in countries such as the Ivory Coast, Ghana, and Eastern regions of Tanzania and Mozambique, where commodity crops are dominant.

Assessing the factors that contribute to deforestation consistently over both space and time using remote sensing data is difficult, particularly when relying on medium-resolution satellite imagery, with a spatial resolution of 10 to 30 meters, which is required for comprehensive worldwide coverage^18^. The difficulty stems partially from similarities and differences, both in spectral and spatial aspects, between land-use practices^4,19,20^. Land uses over large scale are spectrally heterogeneous, with substantial variation in spectral signatures across space, time, elevation, soil types, forest types, and disturbance intensities. These land-use similarities and differences, along with variation in spectral reflectance and phenology across geographic regions, as well as persistent tropical cloudiness, make it difficult to distinguish land uses using satellite imagery consistently. Although variability of land-uses has constrained its classification at larger scales^21^, maps based on automated classification of remotely sensed imagery have successfully monitored drivers of forest loss at regional scales (for example^4^). However, the spatial, temporal, and thematic detail of the regional maps of land-use after deforestation is limited, and varies widely across different geographies and land-use types.

To tackle these challenges, we undertook a continental assessment of the direct drivers of forest loss here, defined as a human-related land-use conversion or land-use following deforestation. We focused on the tropics (30° N to 30$^{\circ }$ S) due to the widespread conversion of natural forest to agriculture, mining, settlements, and commodity tree crops across tropical latitudes and high rates of potential carbon sequestration from tropical tree regrowth. We aimed to use high-resolution remote sensing data^22^, deep learning^17^, and active learning (AL)^23,24^ to accurately identify and map land use following deforestation and assess the trend and hotspots of land-use conversion across countries, and regions in Africa^25^.

## Results

### Active learning for improving land-use classification

**Figure 1:**
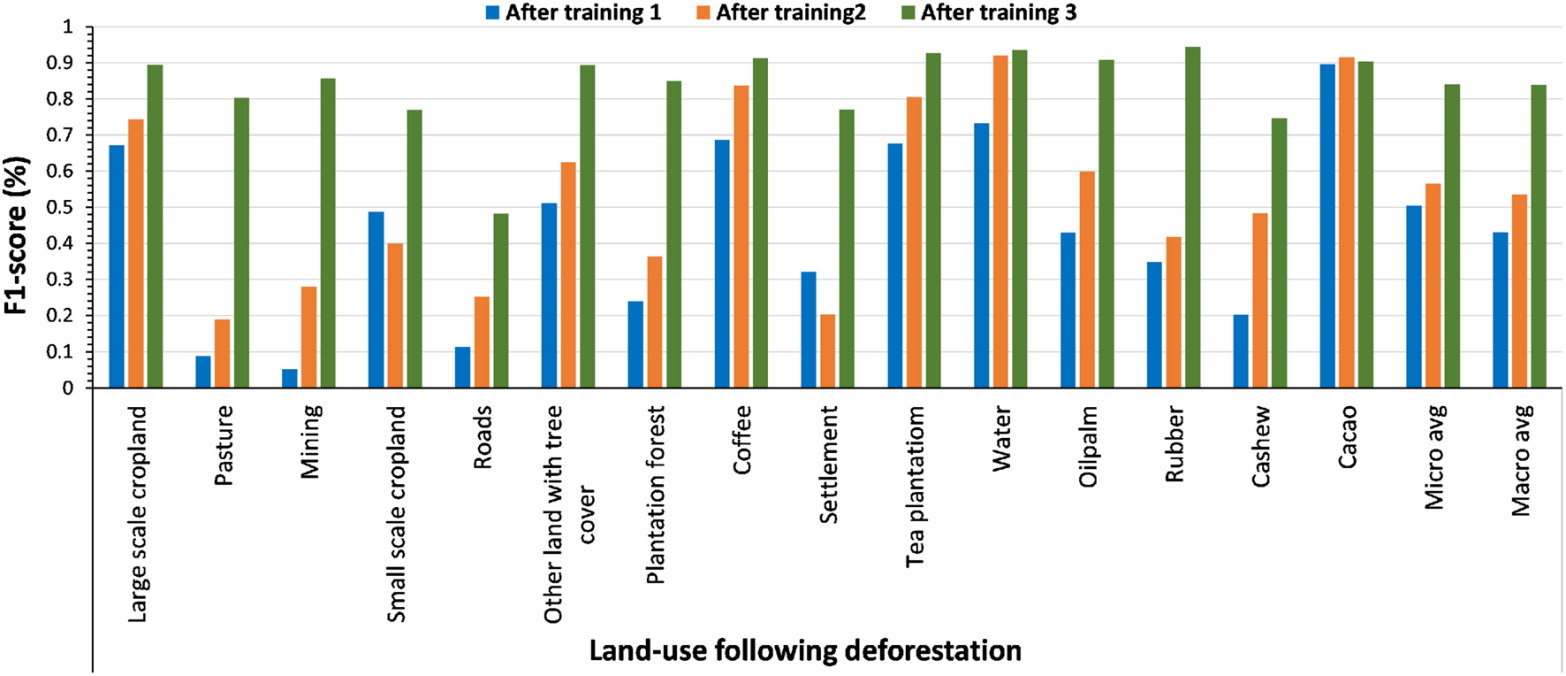
The bar chart shows the performance of the attention U-Net model in classifying land-use following deforestation over three active learning cycles in Africa.

AL could be leveraged to combine heterogeneous data sources with limited labels for the task of semantic segmentation^23,26^. Here we present the results with and without using AL to map land-use following deforestation at a continental scale. The original reference labels are limited, geographically scattered, heterogeneous and are subject to errors (land use delineation error, mix of different land use in one polygon). Using independent test samples stemming from AL, on two AL rounds, we improved the classification performance on all land-use classes. Specifically, the macro average F1-score improved from 43% with the original data to 54% after the first AL round and to 84% after the second round (Fig. 1). The exception to this trend is cacao, which shows high accuracy on all iterations (Fig. 1).

### Generating land-use map prediction at scale

**Figure 2:**
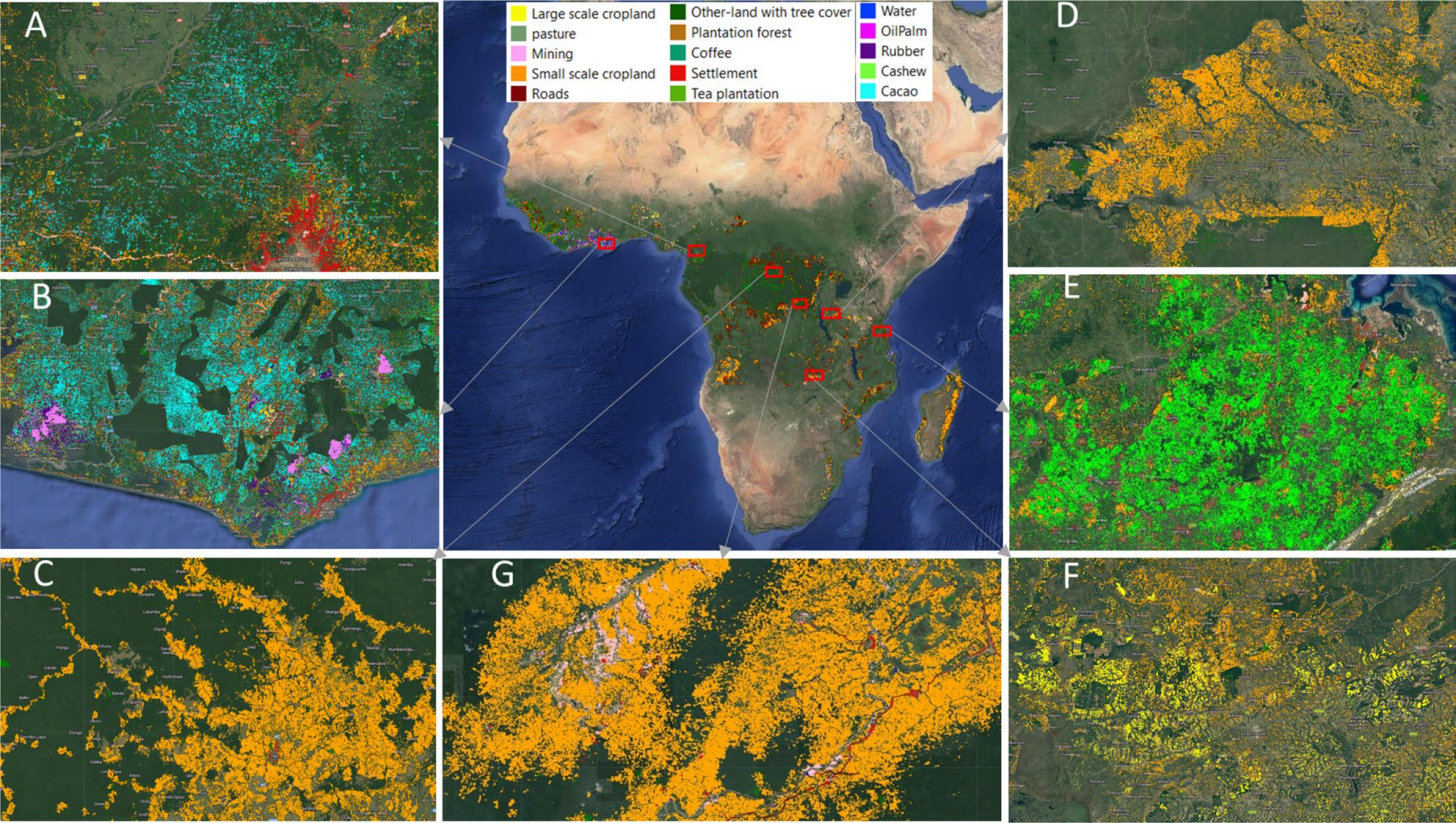
A 5 m resolution land-use following deforestation map in Africa (30$^{\circ }$ south and 30$^{\circ }$ north) corresponding to 2020 using planet-NICFI images and Hansen forest loss data between 2000 and 2020 as proxy for forest loss. The zoomed-in maps show (**A**) cacao expansion in Cameroon, (**B**) cacao, oil palm, and rubber expansion in Ghana, (**C**) small-scale cropland expansion in the DRC, (**D**) small-scale cropland expansion in Tanzania, (**E**) cashew expansion in Tanzania, (**F**) a mix of small-scale and large-scale cropland expansion in Zambia, and (**G**) shows a mix of mining along rivers, roads, and small-scale cropland in DRC for the same period.

**Figure 3:**
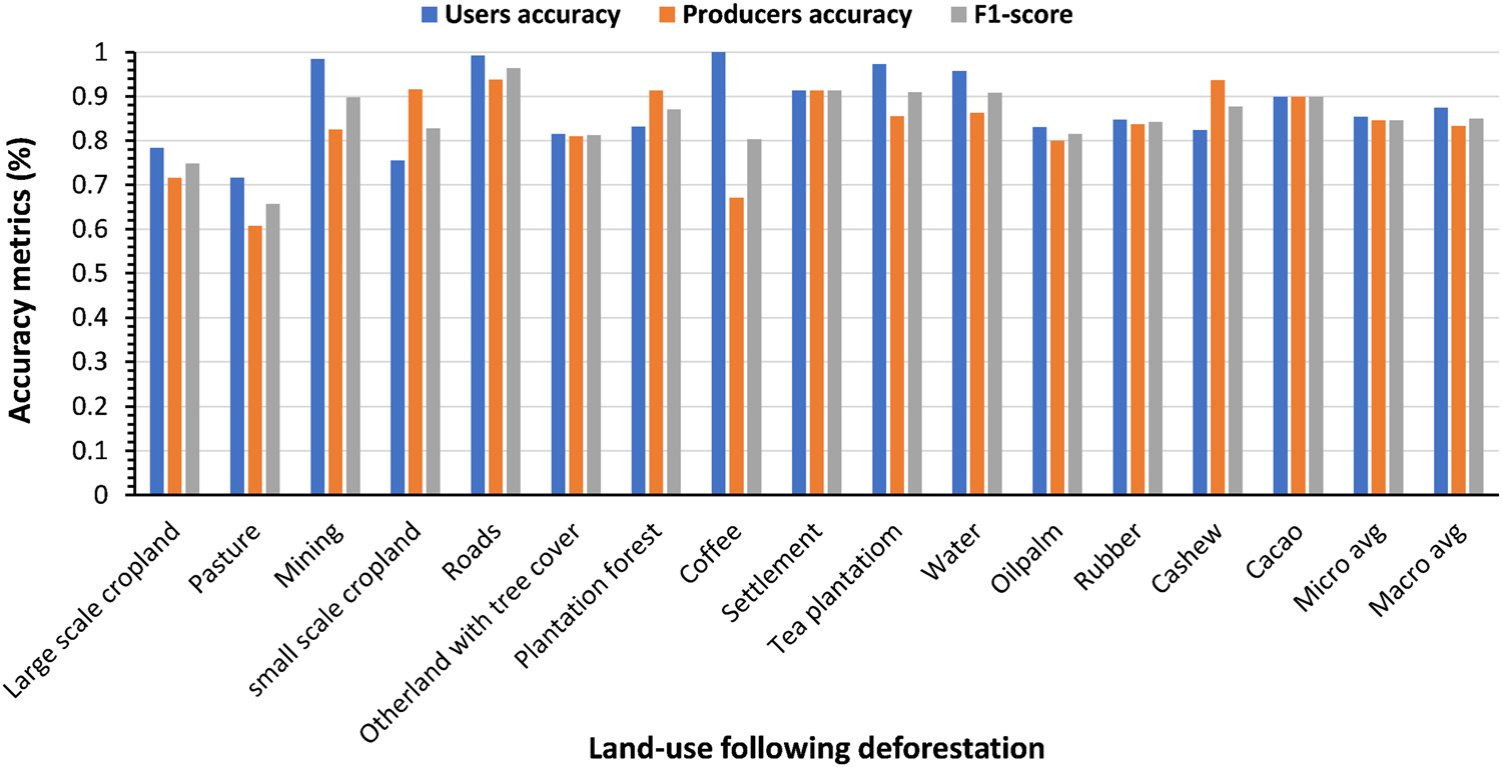
The users, producer’s accuracy and F1-score of the wall-to-wall map generated in (Fig. 2).

We present the first wall-to-wall map of land-use following deforestation across Africa covering forest loss from the year 2001 to 2020 (Fig. 2). The output predicted map is available at a spatial resolution of 5 m, with 15 land-use classes. The map has a users accuracy of 85%, producers accuracy of 84%, and F1-score of 85%  (Fig. 3). For detailed visualisation, the predicted output map of land use following deforestation is available at this website: https://robertnag82.users.earthengine.app/view/africalu.

### Proportion of land-use following deforestation by country

**Figure 4:**
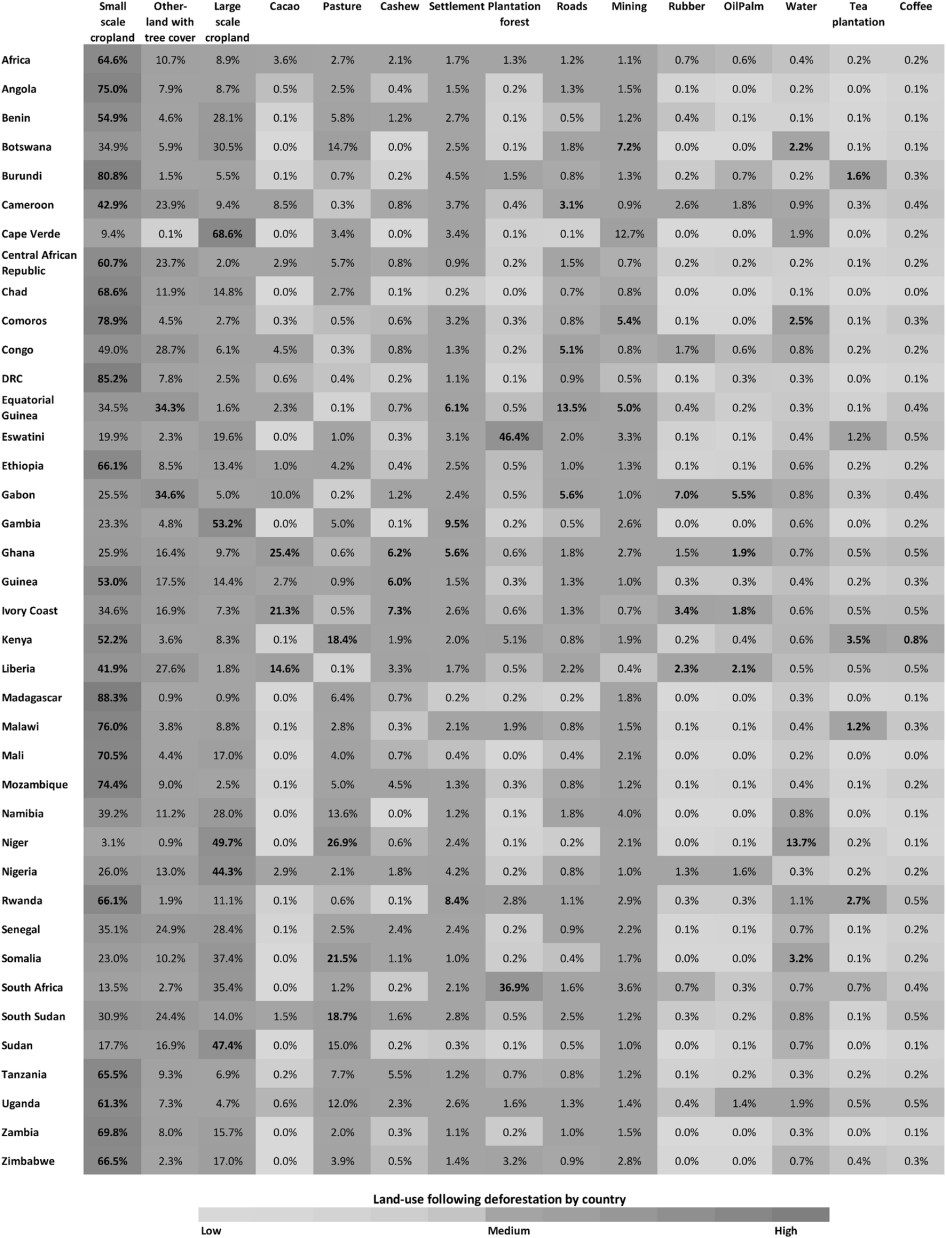
Proportion of land-use following deforestation 2001–2020 by country across continental Africa 30$^{\circ }$ south and 30$^{\circ }$ north.

Small-scale cropland was the dominant driver of forest loss in Africa, resulting in 64% of total forest loss from the year 2001 to 2020 (Fig. 4). This was also the case for most countries, regardless of their contribution to total forest loss, a notably high proportion of small-scale cropland was observed in Madagascar (88%), followed by (85%) in the Democratic Republic of Congo (DRC), Burundi (81%), Comoros (79%), Malawi (76%), Angola (75%) and Mozambique (74%). Other-land with tree cover (OLWTC) was the second highest driver of forest loss in Africa and contributed to 10% of all forest loss in Africa. The highest proportion was observed in Gabon (34%) and Equatorial Guinea (34%). OLWTC constitutes all forest conversion related to fire, windthrow, lightning, speculative clearings, abandoned croplands, and regrowth^6^. Large-scale cropland was the third highest driver of forest loss in Africa (9%), with the highest proportions by country found in Cape Verde (67%), Gambia (53%), Niger (50%), Sudan (47%), and Nigeria (44%). Likewise, the highest proportion of tea plantation establishments was observed in Kenya (4%) and Rwanda (3%).

The proportion of forest conversion for commodity crops such as cacao, cashew, oil palm, rubber, and coffee accounted for 7% of all forest loss in Africa. By country, the highest proportion of cacao was found in Ghana (25%), Ivory Coast (21%), and Liberia (15%); cashew in Ivory Coast constituted (7%), Ghana, Guinea, and Tanzania each constituted (6%), and Mozambique (5%); on the other hand a high proportion of oil palm was found in Gabon (6%), with Liberia, Ghana, and Ivory Coast each having (2%); a high proportion of rubber was mostly found in Gabon (7%), Ivory Coast and Cameroon were having each (3%), and Liberia (2%); the contribution of coffee was mostly found in in Kenya (1%). Additionally, the highest proportion of pasture was observed in Niger (27%), Somalia (22%), and Kenya (18%).

The conversion of forest settlement was mostly observed in Gambia (10%), Rwanda (8%), and Equatorial Guinea (6%); similarly, roads constituted a majority of forest loss in Equatorial Guinea (14%), Gabon (6%), Congo (5%), and Cameroon (3%), while mining had a higher proportion in Cape Verde (12%), Botswana (7%), and Equatorial Guinea (5%). Water was mostly observed in Niger (14%), with most changes associated with meandering rivers. Not surprisingly, the highest proportion of plantation forests was found in southern African countries such as Eswatini (46%) and South Africa (37%).

Additionally, in appendix A we add a table that shows total area of land use following deforestation in Mega hactare (Mha) per land use and per country.

### Trend of land-use following deforestation in Africa

**Figure 5:**
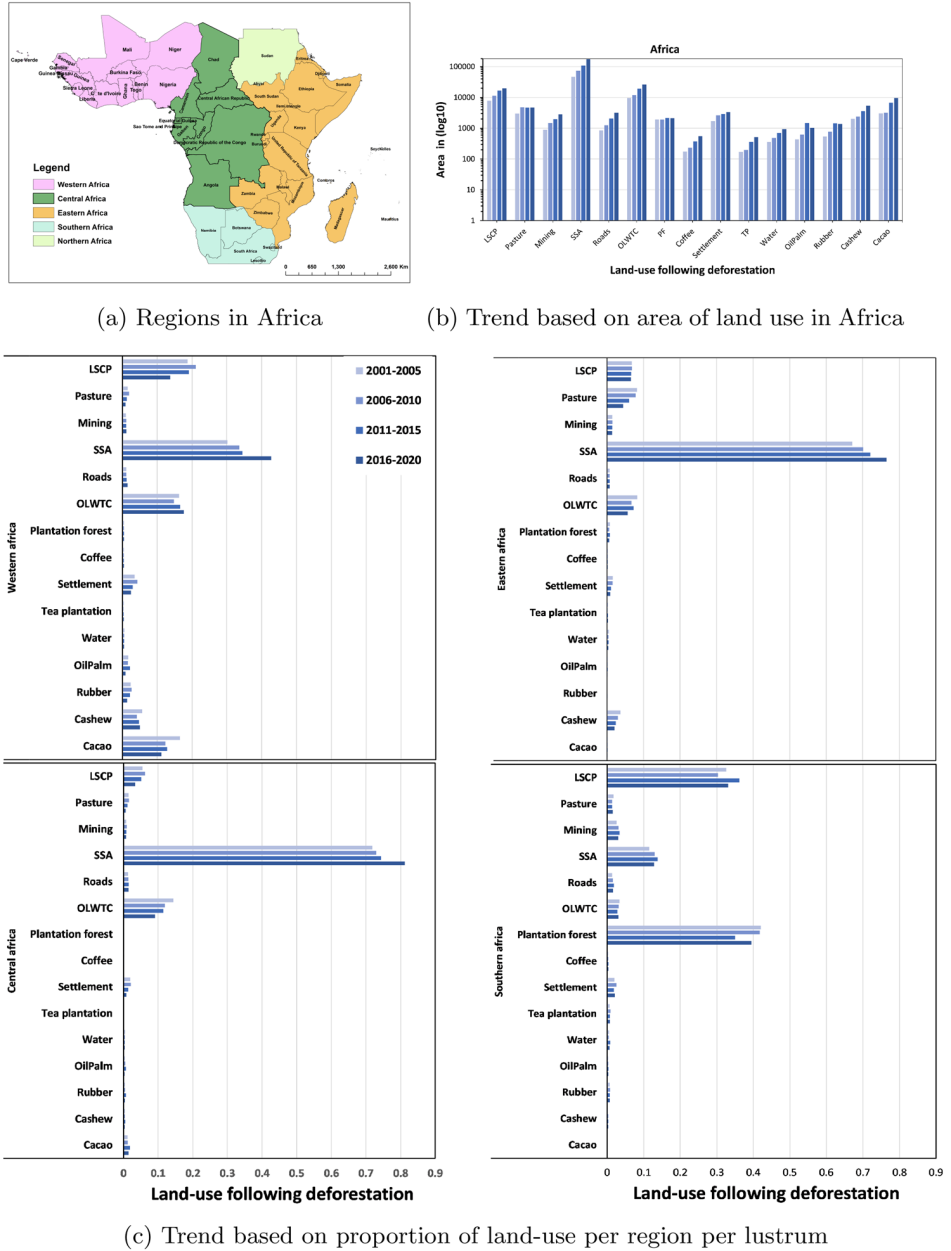
Trend of land-use following deforestation in lustrum from 2001-2020 across four regions of Africa, shown in (**a**) are corresponding regions, in (**b**) we show the trend based on the area ($log10-scsale$) for the entire continent and in (**c**) based on the proportion of land-use following deforestation per region per lustrum. LSCP and SSCP stands for large-scale cropland and small-scale cropland, while OLWTC stands for other-land with tree cover.

Having predicted the land-use following deforestation across Africa for the year 2001 to 2020, we attempted to estimate the trend of the land-use for the entire study area and across four regions in Africa (Fig. 5a) based on area and proportion per lustrum. These regions are western, central, eastern, and southern Africa. Our results suggest an increasing trend in the area of all land-use following deforestation, with the exception of pasture and plantation forest (Fig. 5b). However, when the trend was calculated based on the proportion of each land-use per lustrum, only small-scale cropland showed a positive increasing trend in three regions of western, central, and east Africa with the exception of southern Africa region (Fig. 5c). This show that although the area of almost every land-use is increasing per lustrum, the change is not proportionate.

### Hotspots of land-use following deforestation in Africa

**Figure 6:**
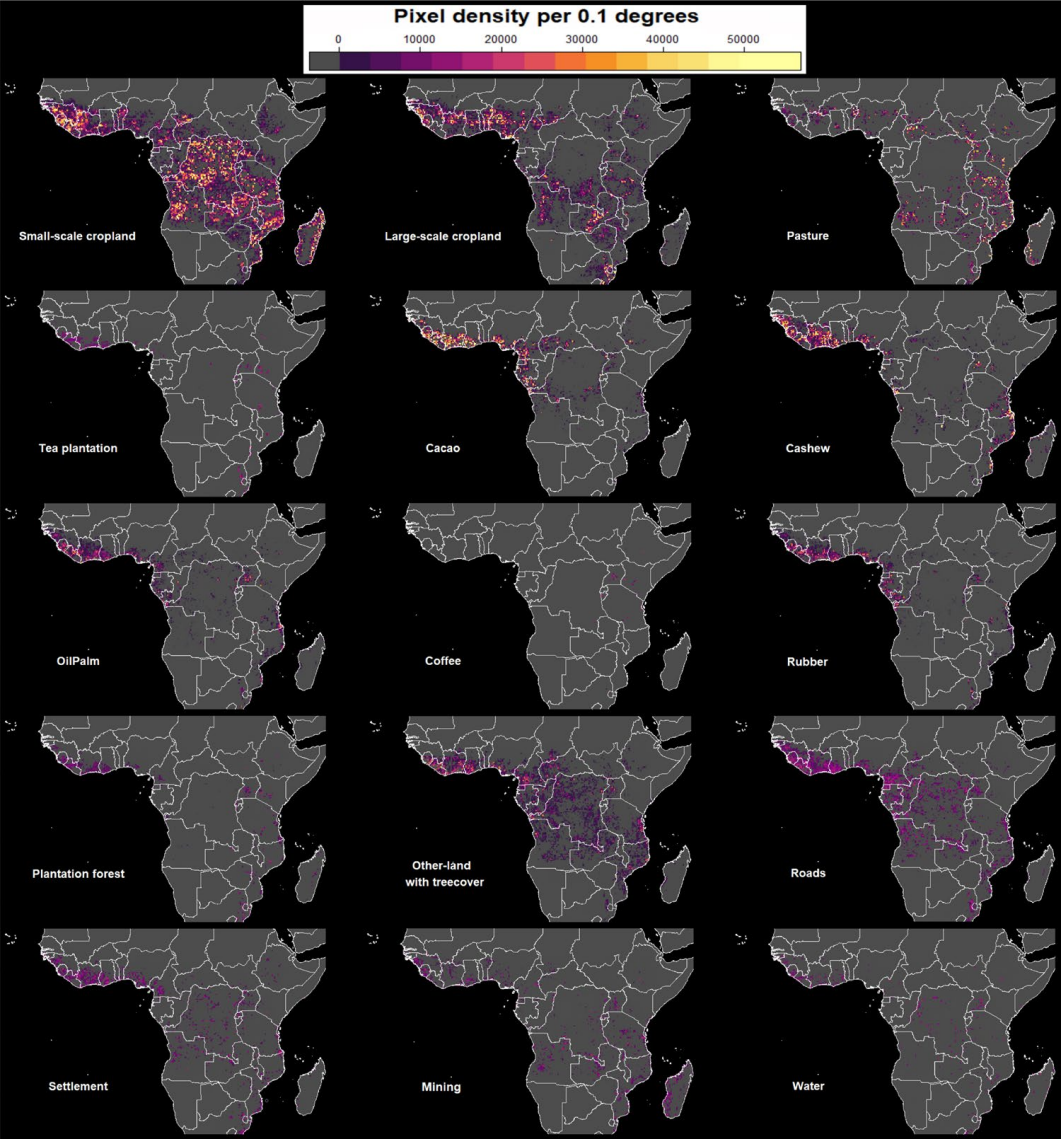
Hotspots of different land-use following deforestation in Africa along 30 $^{\circ }$south and 30 $^{\circ }$ north, for the year 2001 to 2020. The colorbar shows the estimated pixel density of each land-use in a given location.

We observed a considerable spatial variation of hotspots of land-use following deforestation across continental Africa (Fig. 6). The major hotspot locations are small-scale cropland in the Democratic Republic of Congo (DRC), Angola, and Madagascar; large-scale cropland in Nigeria and Zambia; pasture across east Africa but mainly in Tanzania; a major hotspot for cacao is largely along the southern regions of west Africa, but also in central Africa, and form what we call the *arc of cacao*, (see Fig. 6). We also observe hotspots for cashew in northern and central regions of Ivory Coast, Ghana, east-southern Tanzania, and northern Mozambique; hotspots for oil palm were highly observed in Ivory Coast, Ghana, Liberia, Cameroon, and Uganda; coffee in Kenya, Ethiopia; tea plantations in Kenya, Rwanda, and Malawi; rubber in Ivory Coast, Ghana, Liberia, Cameroon, and Gabon; plantation forest in South Africa, and Eswatini; roads in Ghana, Cameroon, Liberia, and Equatorial Guinea; settlements in Ivory Coast, Liberia, Nigeria, and Cameroon; mining in Ghana, Angola and parts of Eastern DRC; hotspots for water were mostly observed along meandering rivers of the coast of Ivory Coast, Ghana, DRC, and along the coast (islands) of Uganda. Of most importance, we observe commodity crops hotspots dominating in western and central African regions and partially forming an arc along the coast of the Atlantic ocean (Fig. 6). In general, these hotspot maps conveys an eye catching message to forest conservation agencies, decision and policy makers by showing the exact causes and locations of forest loss, for which more effort need to be directed and prepare best strategies for mitigation actions for future forest conservation.

## Methods

### Study area

**Figure 7:**
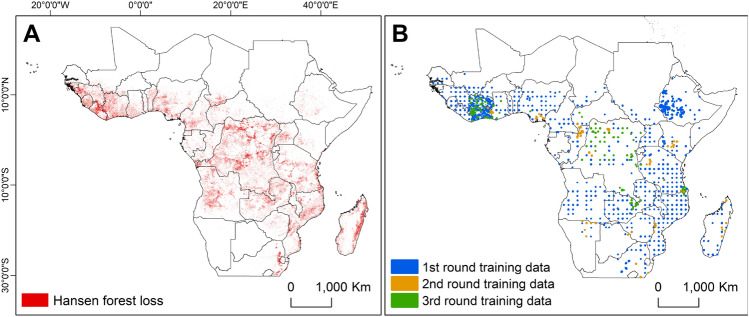
Map showing study location in the African continent along 30$^{\circ }$ south and 30$^{\circ }$ north. (**A**) is the study area map with forest loss locations between 2001 and 2020, and (**B**) shows the locations of the training data over three active learning training cycles.

This study was carried out within the African continent along 30$^{\circ }$ south and 30$^{\circ }$ north, which covers countries in western, central, eastern, and southern Africa (Fig. 7) . The region is characterized by humid forests as well as dry forests. The study area was defined based on the coverage of high-resolution Planet-NICFI data^22^.

### Data

#### Reference land-use data

We identified 15 land-use following deforestation classes as the main direct drivers of deforestation in Africa using information from^5,9,15,17,21,27^. The land-use data from^5^ was obtained via crowd-sourcing using citizen science while^17,21^ annotated the land-use data using high-resolution images in collaboration with stakeholders on the respective country. The data from^15,27^ is based on the FAO global Remote Sensing Survey for 2010, where they used a systematic sampling along latitude and longitude with grids spaced 10km by 10km. Other reference data was retrieved from open source data available via online searching^28–31^. The land-use classes identified and annotated for this study are small-scale cropland, large-scale cropland, pasture, mining, roads, other-land with tree cover, plantation forest, coffee, settlement, tea plantation, water, oil palm, rubber, cashew, and cacao (refer appendix D for class definitions). However, it is important to highlight that the majority of reference labels are available as point vectors, or their polygon contains a mix of land-use classes and does not accurately delineate the land-use borders, making it challenging for direct use in the semantic segmentation task. Thus, extra annotation was implemented using an AL process described in Sect. "Models and implementation details".

#### Satellite imagery

We used annual mosaics of high-resolution planet-NICFI images with 4.77 m $$\approx $$ 5 m resolution to train a deep learning model and map drivers of deforestation in continental Africa (30 $$^{\circ }$$south and 30 $$^{\circ }$$ north)^22^. The image mosaics are made available as a result of Norway’s International Climate & Forests Initiative (NICFI) program, which aim to help protect forest and biodiversity and reduce the impact of climate change^22^. The images used to create the mosaics are acquired from the Planet company, which operates a low-orbit constellation of satellites with a revisit frequency of 1 day. The images are accessible as biannual mosaics from December 2015 to August 2020 and as monthly mosaics from September 2020 onwards. The image mosaics come at a spatial resolution of 4.77 m and have low cloud cover, thanks to the daily acquisition frequency, which has proven useful for forest monitoring over the whole of the tropics. The mosaics consist of four spectral bands, namely blue, green, red, and near-infrared (nir). Since the image mosaics are created from a combination of many sensors and cover various regions in Africa, they have variability in spectral and visual appearance. To correct for this, all the images were normalized such that the range of pixel values is between 0 and 1. This was done for blue, green, red, and near-infra-red bands. Additional vegetation indices such as the normalized difference vegetation index-(NDVI) given as $$ndvi=(nir$$ − $$red)/(nir+red)$$, soil-adjusted vegetation index − (SAVI) given as $$savi=(nir-red)/(nir + red +0.5))*(1.5)$$, and the normalized difference moisture index - (NDMI) given as $$ndmi=(green $$-$$ nir)/(green + nir)$$ were created to enhance models’ capability to segregate land-use following deforestation^17,21^. The inclusion of these indices was considered following results from the test experiments and literature^17,21^. Adding these indices improved the deep learning model performance in identifying land use following deforestation when compared to solely using the typical four bands (blue, green, red and near infra-red) from Planet-NICFI. In addition, in the literature^20,32–35^ the NDVI, SAVI, and NDMI are the most widely used indices for forest change detection, crop phenological monitoring, moisture content, and for land cover classification making them the best possible additional bands for this task. Also, since we only have four bands from Planet-NICFI images, these are the best possible uncorrelated indices we can derive from the data.

### Data preprocessing

**Figure 8:**
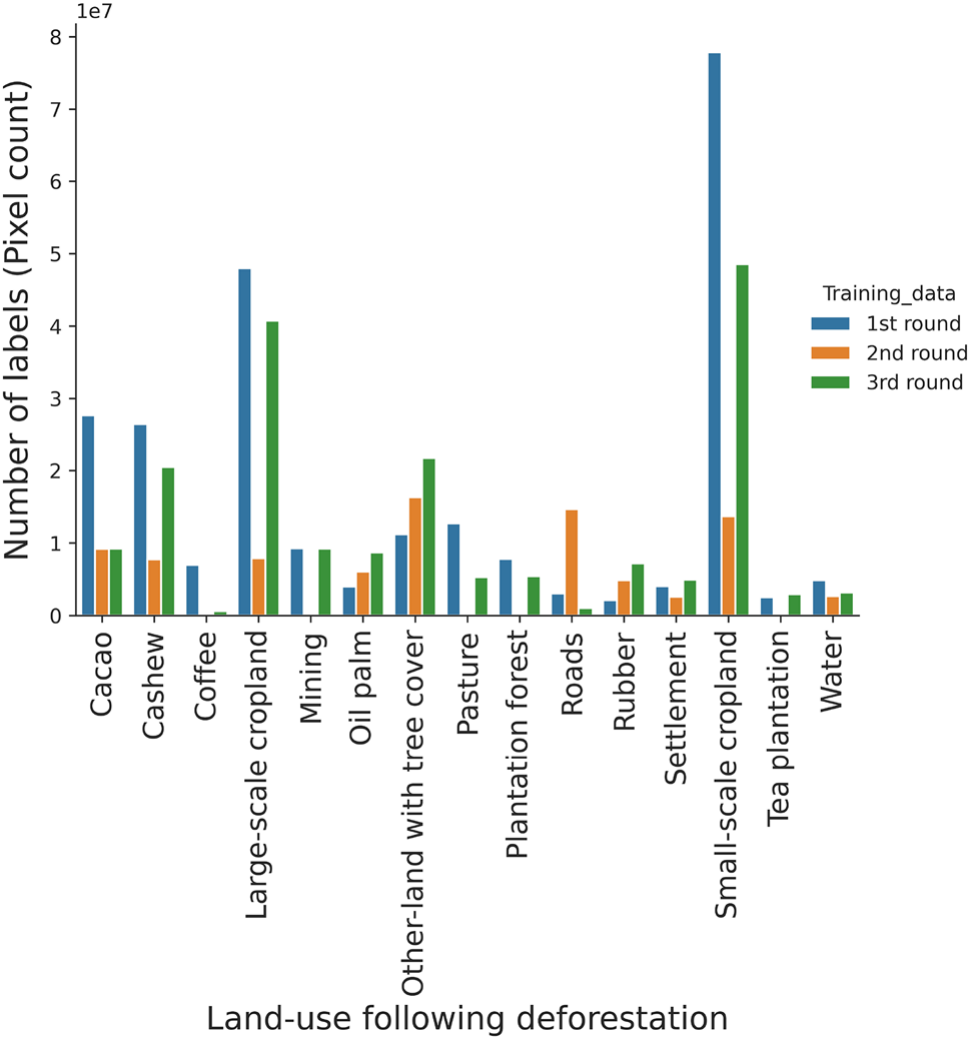
Shows the number of labels in pixel counts used for the model training in the 1st, 2nd, and 3rd rounds of active learning.

**Figure 9:**
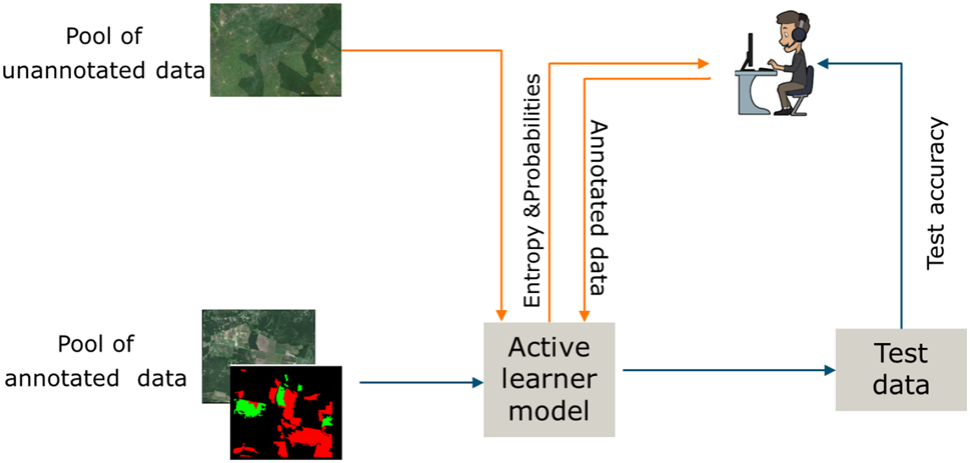
The process of active learning with a pool of annotated data for initial model training, a pool of unannotated data for iterative labeling, and test data for independent testing.

For this study, we created three data pools: (1) a pool of annotated training data, (2) a pool of unannotated training data, and (3) independent test data (Fig. 9). In total, we had 2357 images acquired from all across Africa (refer Sect. "Data", of which only 895 images were having annotations. 80% of the 895 images were placed in a pool of training data, while 20% was left out as independent test data. The remaining 1462 images were placed in a pool of unannotated training data. The unannotated data was later used in Sect. "Models and implementation details" in an active learning cycle where model uncertainty was used to decide which images are useful for improving the model performance. The distribution of land-use classes for initial model training, second, and third active learning iteration is displayed in Fig. 8.

### Research design

In this study, we used high-resolution (4.77 m) satellite images from Planet-NICFI to characterize the spectral, temporal and spatial patterns of land-use following deforestation. We used the Hansen forest loss dataset between 2001 and 2020^2^ as a proxy for forest loss to estimate land-use following deforestation. Based on this dataset, we selected images $\ge 3.8$ ha in size with detected forest loss in the period covered by the dataset (n = 1821). Over each resulting patch containing forest loss pixels, we extracted Planet-NICFI imagery corresponding to the year 2022 and created reference labels via visual interpretation. We then used these labeled training data and deep learning to predict land-use for 2022 as a proxy for the driver of deforestation from 2001 to 2020. The output was a 5 m resolution classified map of land-use following deforestation.

Additionally, we assessed the spatial hotspots and temporal extent of each of the land-use following deforestation across Africa (30 $^{\circ }$ north and 30 $^{\circ }$ south) based on the output map predicted using the planet-NICFI data.

### Models and implementation details

In this study, we adopted the Attention U-Net model developed by^17^ for the classification of land-use following deforestation in Ethiopia, since this architecture had provided high performance in identifying drivers of forest loss from satellite data. The model was used to upscale the characterization of land-use after forest loss in Africa in high thematic detail (fifteen classes) and spatial coverage (continental Africa and Madagascar (30 $^{\circ }$ north and 30 $^{\circ }$ south), as opposed to nine land-use classes and the national scale for which the base model was developed. Although the model had shown to perform well in classifying land-use following deforestation at a country scale, the limited availability of training data and heterogeneity of land-uses across Africa would prevent it from being usable at the continental scale. To counteract this, we incorporated AL in the training process to inform data selection and optimize the labeling process required for the model to learn new and informative features from diverse data sets across regions in Africa. We tested the ability of the attention U-Net model to effectively learn from multiple new data-set across different regions in the continent and be applied to different classes from another set of regions. In addition, we performed an independent assessment of the resulting maps of drivers of forest loss in the whole of tropical Africa.

#### Model details

We trained the attention U-Net model for 200 epochs, with a batch size of 64, using Adam optimization with a learning rate of $10^{-4}$. In order to help dealing with the class imbalance we used the Focal Loss (FL)^36^,

$$FL(p_{i})=-\alpha _{i}(1-p_{i})^\gamma \log (p_{i}).$$

to solve the multi-class segmentation problem. Where $p_{i}$ stands for probability of predicting class i, $\gamma$ for Gamma , and $\alpha$ for alpha. We adopt FL as one of the mathematical function or loss function that is used in a deep learning models to calculate the deviation/error of the predicted value from the true value during the model training process with the goal of reducing this deviation by learning the trainable parameters, such as weights and biases ^36^. There are many loss functions used in deep learning analyses, however focal loss has shown to perform well on data with imbalanced classes^17,36^. During model training the focal loss forces the model to gives more importance to rare/hard classes than majority/easy classes. This is achieved by adding $\gamma$, and $\alpha$, and class weights in a function as modulating factor.

We also performed a padding operation after each convolutional layer in order to ensure that the size of the output stays equal to the input. The feature maps between the convolutional layers were normalized using BatchNorm, and dropout with a rate of 0.1 was used to improve the generalization of the model. These models for classifying drivers of forest loss were implemented and run in the Sepal geospatial analysis platform (SEPAL 2.0)^37^, a cloud-based computing environment offered by FAO, with instance type g8, which provides NVIDIA Tesla M60 GPUs with 32GB of VRAM. The model was implemented using the Keras library^38^ with TensorFlow^39^ as the backend.

#### Evaluation of model performance

The study presents accuracy metrics based on the F1-score, which is calculated as $F1=2(P*R)/(P+R)$^21^. *P* and *F* stand for precision and recall. The class-wise F1-score measures the model’s capability to identify every single class of land-use following deforestation. The average of all classes F1-scores, also known as macro-averaged F1-score, is used to show the overall classification accuracy of the model. This provides the average of all class-wise F1-score values and compensates for any class imbalance, since it gives the same inportance to all classes, thus increasing the weight of samples from rare classes. While the micro-average F1-score computes the aggregated contribution of all classes by using precision and recall values averaged across all classes, thus putting emphasis on the common classes in the data since it gives each sample the same importance^21,40^.

#### Active learning

One challenge of employing a deep learning methodology is that it tends to require a large amount of data for training^41,42^. In reality, there exists a limited amount of training labels to cover the variability of all land-use classes^41^, and the task of labeling all the required land-use data using satellite imagery on a continental scale can be expensive^26^. Indeed, during initial model training using the annotated data described in Sect. "Data", we achieved an unsatisfactory model classification performance on some of the land-uses on independent datasets, with F1-score of 0.05, 0.1, 0.41, 0.25, and 0.14 for mining, roads, settlement rubber, and cashew, respectively. To be able to improve the classification performance of the model for these land-uses we had to adopt AL^23,24^ to identify images where the model provides highly uncertain predictions to identify the most informative images and annotate more labels from these images as an addition in the training pool.

*Iterative pool-based learning* We first train our model using a set of annotated data and assess its accuracy using the independent test data. The first training is done on what is called a pool of annotated data. We then use the trained algorithm to select a set of images from a pool of unannotated data to be annotated by a human annotator. For image selection, we employ entropy

$$H(Y)=-\sum _{i=1}^{k} p_{i}\log (p_{i}).$$

as an uncertainty measure for unlabeled images, not in the training set. $\sum _{i=1}^{k}$ stands for the sum of images’ possible values, given a discrete class membership *Y* and probability *p* of class *i*. Only images with entropy ($>0.6$) were assigned for manual annotation and then later added to the training pool. The process was repeated two times until the best accuracy was obtained for all the classes. In total, 716 labeled images from the training pool were used in the initial training. In the second cycle using AL, we annotated 372 images which were then added to the training pool for second round of model training, followed by 554 images for the third AL training cycle, Figure 9. The final model performance assessment for each cycle was done on 179 separate independent test data, not in either training pool.

### Wall-to-wall mapping

We used FAO and SURFSARA cloud computing platforms^37,43^ to run inference of the fifteen land-use following deforestation classes across the entire African continent, 30$^{\circ }$ south, 30$^{\circ }$ north^22^. The inference was run on 5 m resolution freely available planet-NICFI imagery using the attention U-Net model trained in Sect. "Models and implementation details". We used planet-NICFI imagery for the year 2022 as a proxy for predicting land-use following deforestation from the year 2000 to 2020. The inference was only applied to forest loss areas identified in^2^.

### Assessment of the wall-to-wall map accuracy

To estimate the number of samples needed for evaluating the map of forest loss drivers, we employed a stratified evaluation of area and accuracy based on^44^. The number of samples and accuracy were calculated based on four lustra (2001–2005, 2006–2010, 2011–2015, and 2016–2020) in order to be able to assess temporal variations in the map accuracy and proportion of forest loss drivers. For each stratum (class), sample estimation weights were calculated based on the area estimation of each driver of forest loss for each time period. The calculated weights were utilized to determine the quantity of samples necessary to evaluate the map’s accuracy for each driver over a five-year period. Afterward, the accuracy of the wall-to-wall map was computed through the use of both the user’s and producer’s accuracy measures.

### Hotspot analysis of the wall-to-wall map

Hotspot analysis is an interesting visualization technique to get insight into the data that can inform targeted actions, resource allocation and decision-making for forest conservation^45^. We used the kernel density estimation technique (KDE) to estimate the hotspot of the sixteen land-use following deforestation classes predicted in our wall-to-wall map. KDE uses a bandwidth of a specified size to estimate the density of pixels of a land-use within a location to create heatmaps for each land-use. By using the KDE package in python^46^, we run a kernel of size 0.1$^{\circ }$ × 0.1$^{\circ }$ to predict the hotspot of each of the fifteen land-use following deforestation classes presented in this study. The output is a smoothed-continuous map of pixel size 0.1$^{\circ }$ × 0.1$^{\circ }$, with each pixel representing the density of a given land-use.

## Discussion

### New approach for large-scale mapping of land-use following deforestation

Our results in Sect. "Active learning for improving land-use classification" achieve generalizability at a continental scale and show that it is possible to map land-use following deforestation at a large-scale with high spatial resolution images (5 m) and high thematic detail. In order to bridge across multiple regional reference datasets we applied AL for iterative training, data selection and labeling^23^. The use of AL was fundamental to improve the performance of the deep learning model to classify land-use following deforestation for continental Africa over the traditional model training approaches (see Fig. 1). Particularly in cases where the training data is limited, heterogeneous or is prone to labeling errors (i.e. shape correctness, a mix of land uses), the model using the original data is not able to generalize across regions. In such cases AL is shown to be required to achieve higher accuracy in the prediction process^47,48^. In our study, the only class that obtains good performance using the original data is cacao, which maintains this high accuracy on the two AL iterations (Fig. 1). The reason for this might be attributed to the fact that the reference data for cacaocame from same region (western Africa), which exhibit similar patterns of cultivation, weather and climatic conditions. Thus there is a lower variability than for most other classes, which were retrieved from almost all geographical regions in Africa, with large variations in climate conditions, topography and soil types.

Similar approach and results have been observed in^48^, but only on a small scale applications. Other, similar improvements using AL are also been reported in^49–54^, emphasizing the importance of having human in a loop (AL) when developing models for land use classification task. We hope these model performance gains, coupled with an iterative process of AL, will complement existing visual interpretation methods, and land use mapping tasks, thus accelerating tracking land-use following deforestation at a global scale on a wall-to-wall and more frequently.

### Effects of training data quality

After the first training on the original data, (Fig. 1), the initial model achieved a macro averaged F1-score of 43%, compared to successive models obtaining 54% and 84% after one and two rounds of addition of training data using the AL approach, respectively. We qualitatively assess the reason for the low performance of the initial model as:

- *Limited representation of training data from all spatial location* In appendix B we show the different data sources used in this study. Here we observe the geographical biases of certain land-uses in the training data. A majority of commodity crops data such as cacao, rubber, cashew were retrieved from datasets in countries from western Africa i.e., Ghana, Ivory Coast, and Nigeria, while data for the remaining land use classes i.e. small-scale croplands, large scale croplands, pasture, oil palm shows a much broader distribution in all regions. Another exception is data for coffee and tea classes which mostly comes from Ethiopia and Kenya.
- *Errors in training data* In appendix C are example tiles showing some level of mismatch of the polygon used in model training. The polygon shows a mix between land-uses, possibly due to temporal mismatches, hence creating confusion for the model during training. These errors are an indication of course delineation of land-use boundaries critical for land-use segmentation task.
- *Limited labels* Lack of enough labels for each class are partially responsible for the low performance of the initial model. From Fig. 8 and appendix B we can also observe the under-representation of some of the land-use classes i.e. coffee, tea, roads, rubber. This problem leads to imbalanced classification results as the model tend to put more weight on the majority classes.

### Land-use following deforestation and its implications for forest monitoring

Our finding on wall-to-wall land-use prediction (Sects. "Generating land-use map prediction at scale",  "Proportion of land-use following deforestation by country") indicates that small-scale cropland is the dominant driver of forest loss in continental Africa. This is similar to findings reported in^4^. A notably high proportion of small-scale cropland was found in Madagascar and DRC. According to ^7^ an increase in population and political conflicts are the indirect cause of increasing forest loss due to small-scale cropland in DRC, while in Madagascar, is solely associated with population growth along the western coast^55^. Interestingly, small-scale cropland was the only land-use class that showed an increasing trend of change in relation to other land uses per stratum from the year 2001 to 2020. This increase was observed in western, central, and eastern Africa. Conversely, however, when we analyze the trend based on area change, the area of every land-use increased per stratum with the exception of plantation forest and pasture. This is due to the fact that plantation forests are cleared and re-planted on rotational bases, mostly in exact same locations, which may result in false positive detections of forest loss by the algorithms used in^2^. While for pasture, this might be related to confusion with other-land with tree cover during the classification process.

Forest conversion to commodity crops was another important direct driver of forest loss in Africa (Fig. 6). Using the kernel density estimation method, we identified distinct hotspot patterns of commodity crops in areas of western and central Africa, specifically cacao, cashew, oil palm, and rubber, with other hotspots for cashew in Tanzania and Mozambique. According to^56,57^, the favorable climate condition is the reason for the increasing expansion of commodity crops in these locations. Conversely, land-uses such as tea plantation, coffee, and pasture dominates in east Africa^58–60^. Its proximity to the equator creates favorable conditions for these land-uses^61^. For example, the increase in the establishment of tea and coffee plantations in Rwanda, Kenya, Ethiopia, and Uganda ensures an all-year-round supply of fresh tea to the global markets, which would be challenging in other regions^58–61^. Previously these commodities were for export; however, the current increase in domestic consumption has created demand within the region, thus the need for more plantations^62^. On the other hand, east Africa is known to host nomadic communities with animal grazing/pasture as their main land-use activity^63–65^. Specifically, Ethiopia, Tanzania, and Kenya are known to have a large number of cattle per household^65^. However, the lack of grazing areas and water due to drought and global warming has forced the pastoral communities to move to forested areas where they can get forage for their cattle as well as an opportunity to diversify their practices by farming (silvopastoral)^64^. Thus putting more pressure on remaining forest. This is commonly observed in Ethiopia, Kenya, and Tanzania.

Another highlight is the number of access roads and settlements detected, mostly in western and central Africa (Fig. 6). Growth in the number of roads and settlements in Africa is closely linked to the expansion of agricultural activities^56,66,67^. Specifically in west Africa, the increase in commodity crops has caused an increase in settlements and roads, which are essential to provide accommodation and accessibility for farming communities^56^. Conversely, in central African countries, a majority of newly developed roads are associated with an increase in logging activities (Congo basin)^68^. However, our analyses also indicate that logging roads disappear with time as a result of abandonment and regrowth. Similar studies report an increasing number of roads in central Africa linked to logging activities^68^. Additionally, mining is most prominent in Ghana, eastern to southern DRC, and Angola, with artisanal mining as the main driver of forest loss along river lines of Ghana and eastern DRC (Fig. 6). The presence of mining along rivers has caused not only the loss of forests and wildlife habitat but also a decrease in the quality of water^69^. This poses a health problem to surrounding communities as they become exposed to a toxic chemical used for extracting minerals. Thus a successful forest conservation action would not only save forests but also save communities from health hazards posed by being exposed to mining activities.

*Institutional involvement in the expansion of commodity crops*

As indicated above, in our analysis, western African countries, specifically Ghana, Ivory Coast, and Liberia, have the greatest rate of forest conversion for commodity crop production. In Western Africa, the growth in the production of commodity crops such as cocoa, cashew, oil palm, and rubber is attributable to unique climatic conditions^56,57^. During colonial authority, cashew, cocoa, and rubber were introduced, and seedlings were brought from Latin America^56^. Forest zones were the primary producers of cocoa, rubber, and oil palm, whereas savannas were suitable for groundnut. During and after the fall of the Atlantic slave trade, slave labor played a crucial role in the rise of commodity crop production in many regions of Western Africa^70^.

These commodity crops fared remarkably well in West Africa because millions of smallholder farmers were able to manage their fields using short-term intercropping and intercropping depending on space and soil fertility to respond to the growing demand for commodity crops and poor soils^71^. Several African research and extension institutions, such as the West Africa Agricultural Productivity Program (WAAPP), the Cocoa Research Institute of Nigeria (CRIN), the Rubber Research Institute of Nigeria (RRIN), the Nigerian Institute of Oil Palm Research (NIFOR), and the Ghanaian Ministry of Food and Agriculture (MOFA), have been established in order to address constraints in the production and supply of cash crop seeds and seedlings and to provide credit facilities^71,72^. The primary objective of genetic modification initiatives in Africa has been to raise the agricultural yields required by African consumers and producers of commodity crops. The increased cultivation of these crops, however, has come at the loss of tropical forests.

The “commodity crop revolution” brought up new geographical disparities and increased existing ones, which resulted in bigger migratory flows than ever before^70,73^. Population migrations have a long pre-colonial history related to slave raids as well as free cyclical migration^74^. Even though many people moved from Nigeria to Ghana, the vast majority of them worked as farmhands in colonial Nigeria. At the time, Nigeria was experiencing a rise in exports of cocoa beans from the southwest, oil palm from the southeast, and groundnuts from parts of the central north. Ghana was one of the primary destinations for these migrants^73^. During the dry season of 1952–53 in Nigeria, some 190,000 migrants were recorded moving southward from the northern part of the country. In addition, research has demonstrated that the cultivation of commodity crops has a significant positive effect on household income by alleviating poverty in many African communities, and this has an effect on household migration decisions in many west African countries, including Burkina Faso, Ghana, the Ivory Coast, and Nigeria^73,75^. Due to infrastructure development, rising cash crop output (coffee, cocoa, groundnut), mining sector growth, and the exploration of crude oil, the area has seen an increase in labor migration^74,76^. Many people from the Sahel, including some with their families, moved to commodity crop farms in Ghana, Senegal, Côte d’Ivoire, and Nigeria. Ghana and Côte d’Ivoire largely drew Malians, Chadians, Burkinabes, and Nigeriens to their cocoa plantations, while Senegal and the Gambia supplied labour on their cotton and groundnut farms^73,76^.

### *Comparison with related land use following deforestation work*

Compared to previous works on land use following deforestation^4,7,9,17,77–79^, we advance the land use assessment in multiple aspects. For example,^4^ assessed land use following deforestation on a global scale at a 10 km $\times$ 10 km resolution and on six classes, while ^9^ assessed oil palm plantation at 30 m resolution, and^78^ used U-Net model to map four classes of land use following deforestation in Indonesia. The originality of this paper resides on the fact that it goes beyond traditional benchmarking tasks on mapping land use following deforestation^4,9,78,79^, where either a few classes are accounted for, or are produced at a lower resolution than what would make them useful for both large and local scale applications. This study includes more thematic detail than these previous work, with a specific focus on commodity crops, which dominate in most parts of western Africa and have recently received increased attention for targeted conservation and mitigation actions on deforestation^80^.

Furthermore, this study assesses land use following deforestation at higher spatial resolution (5 m) than previous work on a large scale, making it useful for local scale applications, especially on distinguishing between commodity crops and small-scale/large-scale agriculture, where other studies struggle^4^. For example^4^ estimated shifting agriculture in Africa to account for 92% of forest loss. However, the study overestimates shifting agriculture as it faces the challenge of separating it from commodity crops. This overestimation may be attributed to the coarse scale (10 km $\times$ 10 km) at which the prediction is done. This leads to commodity crops being predicted as shifting agriculture, especially along the southern arc of western to central Africa. Additionally, the algorithms in^9,78^ are methodically akin to the U-Net model used in this work and are closely comparable with our model in terms of architecture used. However, their works maps only a few classes, using Landsat satellite images (30 m), thus hindering a direct performance comparison. This work can thus support a more detailed spatial and temporal assessment of where forests are lost, and the land use activities driving this loss. This will allow the targeting of EUDR and REDD+ mitigation efforts towards specific proximate deforestation drivers in order to achieve more impact.

Another study by^79^ used a sample-based citizen science approach for identification of land use following deforestation, while^7,15,27^ used a sample-based approach to interpret land use following deforestation visually. One drawback of these approaches is that they are not wall-to-wall, and they may miss out on many deforestation drivers in unsampled locations, reducing their usefulness for deriving global statistics or getting a general overview^7,27^. However, these efforts are important for obtaining data useful for developing models used in deriving wall-to-wall maps of land use following deforestation that map every location where deforestation occurred. Specifically, in combination with deep learning and AL, we show that these data sources have great potential to contribute to obtaining maps for monitoring land use following deforestation.

### Limitation and future opportunities

In this study, we used high resolution Planet-NICFI images available for the year 2022 as a proxy for mapping land-use following deforestation from the year 2001 to 2020. The Planet-NICFI data was used because of its high performance when used in detecting subtle changes in land use. However, we acknowledge that the detected land uses on images from the year 2022 might not indicate the primary cause of deforestation in earlier deforestation years. In fact, we might be identifying a secondary land use or regrowth. Thus, care must be taken when using these data to compute statistics of land use for forest loss for the years 2001 to 2015, when Planet-NICFI mosaics were not available. For example, areas where logging roads existed in earlier years, say 2001 to 2015, will now have regrown or be covered by trees in 2022 and hence be classified as other-land with tree cover. Likewise, one specific area can be labeled as commercial cropland in 2022, while being small-scale cropland five years before.

Despite this limitation, this study and derived data fall in line with the recent global efforts to stop deforestation, specifically the recent European Union Deforestation Free Regulation (EUDR) aimed to stop the import of all commodities linked to deforestation after December 2019, including cacao, cashew, coffee, oil palm, wood, soy, and cattle^80^. In addition, it provides a potential pathway for the European Union (EU) as it embarks on achieving its carbon neutrality ambitions by 2050, as set out in its European Green Deal Investment Plan^81^. Importantly, this study showcases the potential of the dataset derived using high resolution satellite datasets and deep learning methods to detect and monitor land use following deforestation, specifically commodity crops, which is critical for the success of the EUDR in an open, transparent, and accessible manner. Likewise, the use of deep learning methods provides an opportunity for repeated monitoring of follow-up land uses on forest loss over successive years.

In this work, we chose to use the Hansen forest loss data as a proxy for deforestation because of its ease of accessibility and download, extensive documentation, including error reporting, single global definition of tree cover, global coverage, relatively high accuracy, and consistency since 2001^2^. Specifically, it is the only consistent large-scale forest loss data that captures loss to some extent in parts of dry forest in Africa, which is the most challenging landscape to track deforestation in. According to a study by^82^, the Hansen forest loss data achieves comparable results to national-derived forest loss data when forest definitions are matched. However, it has also been reported that the Hansen forest loss data does have errors^2,82–85^. Milodowski et al.^84^ reports that small scale disturbances less than 2 ha could be underestimated by up to 50%. Similarly, the Hansen forest loss data in the tropics is reported to have a 13 % false positive rate and a 17 % false negative rate. In Africa, the lowest accuracy was found in sub-Saharan Africa, with false negatives of up to 48%, indicating an underestimation of forest loss in this region, specifically in dry forests^2^. Thus, care must be taken when interpreting this data. Nonetheless, these errors are within acceptable range and may not impact the task of identifying land use following deforestation; on the contrary, the data are useful to gain valuable insight, especially at regional and global scale^2^. We argue that relying on existing deforestation data makes the task of identifying causes of deforestation more tractable without compromising its applicability^3,17^. The recent efforts to integrate different forest loss product provides an opportunity to minimize errors arising from using only one deforestation product^86^

Additionally, despite the success of planet-NICFI data in classifying land-use following deforestation in continental Africa. Persistence cloud cover provided challenges in mapping some parts of the Congo basin and West Africa. We acknowledge the existence of misclassification in parts where planet-NICFI images are covered with cloud cover and haze^34,87^. The inclusion of synthetic aperture radar data (SAR) offers a complementary advantage in detecting land-use in these areas. SAR has the characteristics of imaging through clouds, haze, day and night. It is expected that future missions of SAR data, such as the National Aeronautics and Space Administration (NASA) and the Indian Space Research Organization (ISRO) SAR (NISAR) expected to be launched in 2023^88^ will bridge this gap by providing SAR data continuously in all weather conditions useful for forest monitoring. The NISAR data will be acquired at L-band with a 12-day revisit time, range, and azimuth resolution of 3–10 m and 7 m, respectively. One advantage of L-band SAR is that it can penetrate through a forest canopy, thus useful even in detecting subtle land-uses such as logging roads and artisanal mining in areas where optical data fail to do so due to canopy coverage^89^.

### Supplementary Information

Supplementary Information.

## Data Availability

The reference data are freely available at the citations reference in this Manuscript and upon request to the corresponding author. The satellite data can be accessed via https://www.planet.com/pulse/nicfi-tropical-forest-basemaps-now-available-in-google-earth-engine/. For interactive exploration, the predicted output map of land use following deforestation is available at this website: https://robertnag82.users.earthengine.app/view/africalu.
