# Supplementary material for: Mapping the diversity of land uses following deforestation across Africa
Source: Sci Rep. 2024 Jan 19;14:1681. doi: 10.1038/s41598-024-52138-9 (PMC10798993; doi:10.1038/s41598-024-52138-9)
Supplement: Supplementary file 1 — Supplementary Information. [file 41598_2024_52138_MOESM1_ESM.pdf]

Appendix A Area of land-use following deforestation by country

Here we present a table that shows the area of land use following deforestation in Mega hactare (Mha) including the total area per country in the last column. Presenting the area in Mha is useful for the reader to compare with in-situ statistics and turns the shares into absolute figures. However, we want to highlight that, this data was derived based on the global Hansen deforestation layer as a proxy for deforestation thus it does have errors (underestimation and overestimation of forest loss) [2, 90, 91], especially when computing absolute values [82, 84, 85]. Thus the absolute total area determined from this data will have bias, thus should be used/interpreted with caution. Thats is why to minimize these bias we chose to present the amount of land use following deforestation in proportion (%) (Figure 4), instead of absolute values.

	Small scale cropland	Other-land with tree cover	Large scale cropland	Cacao	Pasture	Cashew	Settlement	Plantation forest	Roads	Mining	Rubber	OilPalm	Water	Tea plantation	Coffee	Total (Mha)
Africa	404549.170	67246.322	55595.827	22488.028	17125.437	13433.335	10553.928	8080.659	7358.839	7159.060	4186.242	3555.611	2484.728	1235.054	1318.852	626371.092
Angola	34598.121	3649.914	4036.557	211.961	1171.274	198.291	708.801	72.831	581.682	703.257	47.304	17.905	85.660	15.792	47.839	46147.188
Benin	3431.569	287.637	1758.046	7.236	363.076	72.532	167.204	7.087	31.709	72.386	23.338	6.804	6.707	3.269	7.510	6246.109
Botswana	34.207	5.786	29.870	0.000	14.419	0.034	2.406	0.133	1.779	7.017	0.001	0.000	2.184	0.052	0.144	98.032
Burundi	433.511	8.300	29.420	0.690	3.519	0.833	24.010	8.251	4.373	7.200	1.289	3.647	1.308	8.398	1.642	536.390
Cameroon	7149.104	3990.821	1567.328	1419.949	54.369	140.228	619.051	69.652	524.237	155.639	435.069	302.606	142.711	46.149	64.305	16681.217
Cape Verde	0.788	0.012	5.758	0.000	0.285	0.000	0.283	0.012	0.012	1.068	0.000	0.000	0.161	0.002	0.017	8.397
Central African Republic	5865.062	2286.549	191.033	282.476	548.779	79.039	86.985	17.445	149.633	69.077	17.084	21.611	17.043	12.750	16.551	9661.116
Chad	5887.584	1022.214	1269.662	0.254	231.259	10.224	19.420	2.451	60.475	68.822	0.467	0.571	11.280	0.920	2.086	8587.689
Comoros	47.873	2.701	1.611	0.171	0.308	0.351	1.919	0.174	0.457	3.265	0.031	0.028	1.486	0.088	0.187	60.647
Congo	4363.313	2556.515	539.769	397.474	22.912	69.151	112.419	20.091	458.517	71.156	151.582	49.193	68.934	14.226	17.089	8912.340
DRC	138777.732	12761.981	4078.973	971.022	611.762	347.079	1720.773	176.537	1436.400	818.413	139.631	435.831	427.056	64.736	160.029	162927.955
Equatorial Guinea	424.643	421.298	20.014	27.876	1.113	8.006	75.102	5.979	165.731	61.529	5.403	2.560	3.558	1.567	4.789	1229.169
Eswatini	262.556	29.818	257.782	0.245	12.521	3.590	40.189	611.562	25.802	43.343	1.840	0.956	4.777	15.692	6.190	1316.860
Ethiopia	4134.494	531.390	840.031	63.480	260.637	25.070	158.869	30.966	60.587	78.506	5.832	7.452	37.041	9.659	10.406	6254.418
Gabon	1179.257	1600.912	232.881	463.104	8.249	57.795	109.088	21.567	259.046	47.765	326.196	255.142	36.969	14.089	17.352	4629.411
Gambia	41.568	8.525	94.782	0.000	8.982	0.124	17.009	0.292	0.892	4.545	0.018	0.013	1.073	0.041	0.303	178.167
Ghana	4550.910	2876.247	1694.818	4452.568	106.372	1085.671	985.070	102.479	315.781	468.848	263.483	332.644	129.659	82.066	95.267	17541.882
Guinea	13769.491	4533.825	3729.352	694.405	233.393	1569.137	387.067	79.242	330.943	250.201	73.171	71.159	116.723	53.483	70.264	25961.856
Ivory Coast	15573.849	7608.919	3272.554	9570.307	239.535	3289.406	1188.290	250.543	588.896	333.494	1537.429	814.459	266.200	208.552	215.860	44958.292
Kenya	2801.912	193.121	447.163	7.928	986.371	104.374	108.453	275.193	43.959	102.560	11.304	22.791	33.748	187.304	45.492	5371.671
Liberia	8094.022	5333.378	348.730	2827.524	22.647	633.671	326.835	101.493	430.176	73.105	444.872	408.397	97.425	87.280	90.204	19319.759
Madagascar	40466.847	406.652	424.552	14.531	2937.466	304.926	109.450	91.938	70.341	808.545	6.611	11.196	123.739	16.769	28.920	45822.484
Malawi	2407.738	119.807	277.766	3.254	88.163	10.679	65.065	61.129	24.706	46.822	2.596	2.389	12.832	36.845	10.010	3169.802
Mali	2555.039	160.443	614.760	0.000	146.112	23.628	15.767	1.639	15.580	77.795	0.759	0.880	9.051	0.955	1.554	3623.961
Mozambique	34327.465	4176.218	1138.715	37.208	2292.926	2089.183	589.939	139.481	376.021	551.070	91.538	48.292	164.296	43.384	94.591	46160.328
Namibia	75.667	21.708	54.070	0.000	26.194	0.000	2.370	0.167	3.508	7.659	0.000	0.000	1.616	0.038	0.150	193.145
Niger	0.169	0.048	2.715	0.000	1.472	0.034	0.130	0.005	0.012	0.116	0.003	0.004	0.747	0.010	0.004	5.468
Nigeria	7620.853	3791.726	12982.006	848.509	626.139	517.278	1242.190	69.243	231.839	305.063	384.180	465.626	81.323	46.180	60.938	29273.093
Rwanda	398.574	11.283	67.221	0.883	3.457	0.663	50.716	17.010	6.630	17.627	1.532	1.572	6.642	16.441	2.787	603.038
Senegal	474.183	335.912	383.897	1.019	33.457	32.454	32.345	2.379	12.777	29.377	0.905	0.960	8.868	1.044	2.181	1351.757
Somalia	52.124	22.992	84.616	0.012	48.605	2.396	2.237	0.475	0.959	3.795	0.085	0.104	7.324	0.141	0.507	226.371
South Africa	1855.470	376.956	4865.100	2.345	165.823	32.222	290.109	5082.945	223.819	493.643	97.246	43.685	90.155	90.593	48.848	13758.958
South Sudan	690.304	543.956	312.226	33.614	418.102	35.074	62.959	11.615	56.797	26.965	5.790	4.373	17.320	3.094	10.379	2232.566
Sudan	13.947	13.309	37.342	0.014	11.792	0.155	0.234	0.072	0.413	0.815	0.006	0.089	0.536	0.013	0.089	78.827
Tanzania	27300.338	3876.482	2882.871	76.647	3230.277	2294.570	498.271	295.276	314.258	506.964	58.737	65.090	131.311	66.809	84.344	41682.245
Uganda	6450.705	763.612	496.860	65.609	1267.421	241.417	273.005	166.579	139.848	151.276	44.247	150.969	203.874	57.556	55.974	10528.951
Zambia	24203.586	2769.860	5440.349	4.805	679.179	121.213	368.714	81.342	356.030	509.230	3.888	4.359	91.629	6.258	26.775	34667.216
Zimbabwe	4234.595	145.496	1084.631	0.909	247.071	32.839	91.187	205.386	54.217	181.102	2.779	2.254	41.765	22.810	17.278	6364.320

Land-use following deforestation by country

Low Medium High

Fig. A1: Area in (Mha) of land-use following deforestation 2001-2020 by country across continental Africa 30 °south and 30 ° north

Appendix B Training, validation and testing data sources

Below in Appendix B2, is a list of sources of data used in this study. The FAO 2010 Global Remote Sensing Survey data comes from [15, 27] with seven

classes of interest for this study (Appendix B2) and covers the whole study area. The data from IIASA [79] also covers the entire study area, however the data comes as point data showing the dominant land use for each location, thus does not delineate the land use boundaries. The Google research open-building dataset also covers the entire study area. Other data sources are either regional-specific and /or contains one land use class. For example [17] contains data from Ethiopia. Data from ICRAF covers only Ivory Coast, while IPIS data [30] contains mining data from the Democratic Republic of Congo (DRC).

Data	LSCP	Pasture	Mining	SSCP	Roads	OLWTC	PF	Coffee	Settlement	TP	Water	OilPalm	Rubber	Cashew	Cacao
FAO 2010 global Remote Sensing Survey	x	x	x	x		x			x		x				
Crowdsourced deforestation drivers (IIASA) (Bayasa et al., 2022) http://pure.iiasa.ac.at/id/eprint/17539/)	x	x	x	x	x		x		x			x			
Masolele et al,. 2022 (Ethiopia)	x	x	x	x	x	x	x	x	x	x	x				
ICRAF, Econometric	x			x		x			x		x	x	x	x	x
NAFORMA (Tanzania)	x		x	x			x							x	
Large-scale farms and small holder (Jann et al,. 2018) (Zambia)	x			x											
Global Map of Oil Palm Plantations (Descale et al., 2021)												x			
Kenya GIS data (World Resources Institute - https://www.wri.org/data/kenya-gis-data)	x			x			x	x		x					
Namibia	X			X											
Ghana	x		x	x								x	x	x	x
Google research open-buildings dataset (https://sites.research.google/open-buildings/)									x						
https://ipisresearch.be/home/maps-data/open-data/ (Mining)			x												
Landuse data Nigeria (https://grid3.gov.ng/datasets)	x			x					x			x		x	x
Satellite based visual interpretation	x	x	x	x	x	x	x	x	x	x	x	x	x	x	x

Fig. B2: Sources of training, validation, and testing data used in this study.

Appendix C Errors in training data

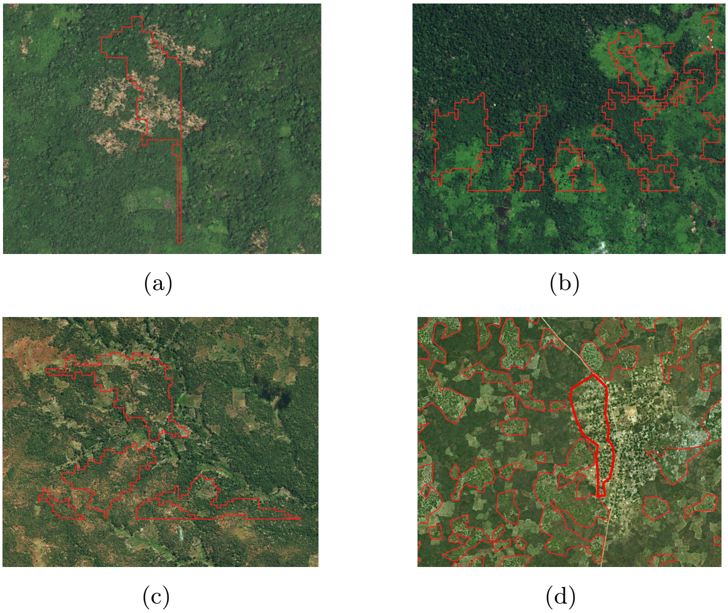

Fig. C3: Examples of errors in the reference labels of the training data. (a), (b) and (c) shows a mix of small-scale croplands and forest edges, while in (d) we observe settlement labeled as cashew crop in the bright red polygon.

Appendix D Definitions of land-use following deforestation classes

Main classes	Subclasses	Sub classes	Description
**Agriculture**	Cacao*	-	• Land under cultivation for cacao
	Rubber*	-	• Land under cultivation for Rubber
	Oil palm*	-	• Land under cultivation for Oil palm, both small scale and large scale
	Tea plantations*	-	• Land under cultivation for tea
	Pasture*	-	• Land used predominantly for grazing; in either managed/cultivated (pastures) or natural (grazing land) setting; includes grazed woodlands
	Coffee*	-	• Land under cultivation for Coffee
	Cashew*	-	• Land under cultivation for Cashew
	Other croplands	Small-scale cropland*	Land under cultivation for crops, characterized by very small (<0.5 ha) to small field sizes (0.5–2 ha)
		Large scale cropland*	• Land under cultivation for crops, characterized by medium (2–20 ha) to large (>20 ha) field sizes
**Plantation forest***	-	-	• Planted forest established for commercial or other related wood extraction purposes
**Infrastructure**	Settlements*	-	•Residential areas or infrastructures built-up for housing, industry, or commercial purposes.
	Roads*	-	•Transport, accessibility
**Mining***	-	-	•Land used for extractive subsurface and surface mining activities (e.g. underground and strip mines, quarries and gravel pits), including all associated surface infrastructure.
**Water***	-	-	• Natural (river, lake etc.) or man-made water bodies (e.g. reservoirs), floods, meandering rivers
**Other land with tree cover***	-	-	• All land that cannot be classified into the above land use categories

Fig. D4: Definitions of each category (denoted by a * symbol) of land-use following deforestation as used in this study.
